# Evaluation of Two Types of Intramedullary Jones Fracture Fixation in a Cyclic and Ultimate Load Model

**DOI:** 10.1002/jor.24530

**Published:** 2019-11-27

**Authors:** Madeleine Willegger, Emir Benca, Lena Hirtler, Maximilian F. Kasparek, Gregor Bauer, Shahin Zandieh, Reinhard Windhager, Reinhard Schuh

**Affiliations:** ^1^ Department of Orthopedics and Trauma Surgery, Division of Orthopedics Medical University of Vienna Vienna Austria; ^2^ Center for Anatomy and Cell Biology, Division of Anatomy Medical University of Vienna Vienna Austria; ^3^ Department of Radiology and Nuclear Medicine Hanusch Hospital Vienna Austria

**Keywords:** Jones fracture, intramedullary screw, fracture‐specific screw, headless compression screw, bone mineral density

## Abstract

Implant choice is a matter of concern in athletes and active patients who sustain a Jones fracture because they are prone to failure including non‐union, screw failure, and refracture. The aim of this study was to compare the biomechanical behavior of a Jones fracture‐specific screw (JFXS) with a cannulated headless compression screw (HCS) in a simulated partial weight‐bearing and ultimate load Jones fracture fixation model. Ten matched pairs of human anatomical specimens underwent Jones fracture creation and consecutive intramedullary stabilization with a solid JFXS or a cannulated HCS. The bone mineral density was assessed prior to testing. Cyclic plantar to dorsal loading was applied for 1000 cycles, followed by load to failure testing. Angulation was measured by an opto‐electronic motion capture system and mode of failure classification was determined by video analysis. Paired analysis showed no statistically significant difference between both screw constructs. Ultimate load reached 236.9 ± 107.8 N in the JFXS group compared with 210.8 ± 150.7 N in the HCS group (*p* = 0.429). The bone mineral density correlated positive with the pooled ultimate load (*R* = 0.580, *p* = 0.007) for all constructs and negatively with angulation (*R* = −0.680, *p* = 0.002) throughout cyclic loading. Solid fracture‐specific and cannulated headless compression screws provide equal ultimate loads and stiffness for Jones fracture fixation. A low bone mineral density significantly impairs the construct stability and the ultimate load of both intramedullary screw constructs. © 2019 The Authors. *Journal of Orthopaedic Research* ® published by Wiley Periodicals, Inc. on behalf of Orthopaedic Research Society J Orthop Res 38:911‐917, 2020

Jones fractures or proximal fifth metatarsal fractures (Zone II and III) are common injuries in competitive and recreational athletes.^1^, ^2^, ^3^, ^4^, ^5^, ^6^ In addition to the high incidence of symptomatic delayed unions and non‐unions when treated conservatively, a long time of immobilization is undesirable for an active patient.^1^, ^2^, ^3^, ^4^, ^5^, ^6^, ^7^, ^8^, ^9^, ^10^ Therefore, a primary surgical approach has been advocated, especially in the athletic population.^11^, ^12^ Intramedullary screw fixation emerged as gold standard treatment with good to excellent clinical results during the last decades.^2^, ^5^, ^8^, ^12^, ^13^, ^14^, ^15^, ^16^, ^17^ Patients benefit from a minimally invasive surgical approach, a more expeditious return to sports and competition, and of higher union rates.^2^, ^4^, ^16^, ^18^, ^19^, ^20^ The aim of surgical Jones fracture fixation in the elite athlete is to enable a fast return to play, which indicates that surgical fixation should withstand an early weight‐bearing regimen. However, certain athletes are prone to failure, possibly due to increased physical demands, repetitive stresses, and inadequate initial fixation. Reported failure of intramedullary screw fixation includes delayed union, non‐union, and refracture associated with screw failure, especially in basketball, football, and soccer players.^8^, ^10^, ^16^, ^21^, ^22^, ^23^, ^24^ Several screw designs have been used to treat Jones fractures. Currently, neither clinical nor biomechanical studies comparing different screw types offer decisive results, resulting in no consensus concerning the ideal implant or screw diameter.^14^, ^17^, ^19^, ^25^, ^26^, ^27^, ^28^, ^29^ Cannulated screws provide an easy insertion technique but might bear detrimental biomechanical behavior compared with solid screws.^25^, ^26^, ^28^, ^30^, ^31^, ^32^ Solid screw insertion can be technically demanding and a prominent screw head may lead to soft tissue irritation.^1^, ^2^, ^3^, ^6^, ^21^

In order to aim for clarification with regard to biomechanical aspects of screw types used in Jones fracture treatment, we compare a solid Jones fracture‐specific screw with a cannulated headless compression screw in a biomechanical Jones fracture fixation model by simulating initial post‐operative weight‐bearing and ultimate loading.

## METHODS

### Specimens and Bone Mineral Density (BMD) Assessment

Ten matched pairs of fresh human foot specimens (four female and six male pairs) were used for this biomechanical study. Donor age ranged from 64 to 92 years (mean 78.8 ± 8.7 years). The specimens were obtained from voluntary donors who consented to donate their body for research and teaching purposes to the Medical University of Vienna during lifetime. Institutional Review Board approval was granted prior to the conduction of the study (EK 2077/2013). The specimens were stored at –80°C and thawed at +4°C 48 h prior to testing to prevent tissue dehydration. Ahead of specimen preparation and biomechanical testing, dual‐energy X‐ray absorptiometry (DEXA) scans of the calcaneus were carried out to determine BMD. Areal BMD measurements were taken with Lunar Prodigy series X‐ray (GE Lunar Prodigy; GE Healthcare, Chicago, IL) and reported as g/cm^2^. Prior studies have reported an excellent repeatability for BMD evaluation using Lunar Prodigy densitometer.^11^, ^26^, ^33^ To minimize potential left/right bias, one foot of each pair was assigned to Jones fracture‐specific screw fixation (JFXS group), and the contralateral foot was assigned to conventional cannulated headless compression screw fixation (HCS group) with an equal number of right and left feet in each group.

### Jones Fracture Creation and Intramedullary Screw Fixation

**Figure 1:**
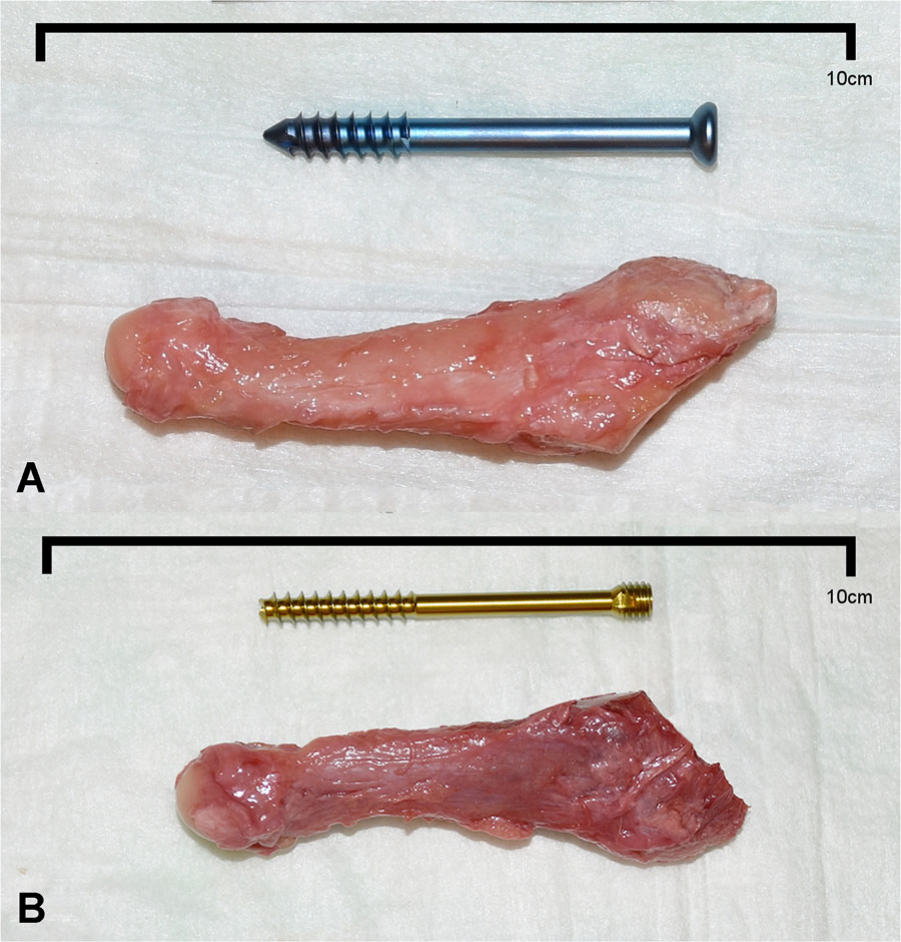
Representative specimens and screws. (A) Right large fifth metatarsal specimen with a 6.0 × 55 mm solid Jones fracture‐specific screw (Jones Screw; Arthrex Inc., Naples, FL). (B) Left small fifth metatarsal specimen with a 4.5 × 50 mm cannulated headless compression screw (HCS; DePuySynthes, Solothurn, Switzerland). [Color figure can be viewed at wileyonlinelibrary.com]

The fifth metatarsals were dissected and disarticulated from the feet (Fig. 1A and B). All specimens proved valid for biomechanical testing by visual inspection to verify intact bone integrity. A padded machine vice was used to stabilize the bone during preparation for intramedullary screw fixation. A longitudinal line was drawn on the metatarsal to check for the rotational alignment during Jones fracture creation and fixation. According to Lawrence and Botte's classification of proximal fifth metatarsal fractures, the Jones fracture (Zone II) is a transverse fracture at the meta‐diaphyseal junction without extension distal to the 4‐5 intermetatarsal joint.^13^, ^26^, ^34^, ^35^ A complete transverse fracture was subsequently created with an oscillating saw at the distal aspect of the 4‐5 intermetatarsal articular facet. The proximal part of the bone was held in place with a small forceps and the appropriate JFXS or HCS was implanted with respect to the “fit and fill” principle.^18^, ^19^, ^36^

The JFXS group received a solid partially threaded titanium screw with a small low‐profile head, especially designed for Jones fracture treatment (Jones Screw, Arthrex Inc., Naples, FL) (Fig. 1A). This screw is available in a diameter of 4.5, 5.5, and 6.0 mm. In our study, we used 4.5 and 6.0 mm screws. Intermediate‐sized screws were not used. Surgical preparation of the intramedullary canal was carried out according to the manufacturer's proposed technique. A 2.0 mm guide wire was inserted “high and inside” by direct visualization and orientation, inspired by previously published anatomical landmarks.^4^, ^5^, ^8^, ^10^, ^21^, ^22^ Afterward a 3.5 mm drill guide was introduced over the guide wire and a 3.5 mm cannulated drill was advanced into the proximal aspect of the fifth metatarsal. Care was taken to avoid penetration of the cortical bone. The manufacturer's cannulated drill and tap have black laser markings for the available screw lengths. During taping the appropriate screw size and length was measured. The required screw length was estimated as about 70% of the metatarsal length and selected by visual inspection superimposing the screw over the metatarsal.^4^, ^5^, ^8^, ^10^, ^12^, ^15^, ^17^, ^19^, ^37^ Care was taken that the screw was not too long, preventing lateral fracture site gapping, but long enough that threads were distal to the fracture site. The intramedullary screw had to “fit and fill” the medullary canal. The tightness of the tap was gauged by direct tactile feedback. If the 4.5 mm tap felt undersized, a 6.0 mm tap was used. In each specimen a good cortical bite of the screw was aimed for. Small specimens received a 4.5‐mm screw and in larger specimens a 6.0 mm screw was implanted.

In the HCS matched pairs, a countersinkable cannulated partially threaded titanium screw (HCS; DePuySynthes, Solothurn, Switzerland) was implanted (Fig. 1B). This screw is available in a diameter of 4.5 and 6.5 mm. Both sizes were used for Jones fracture fixation in our study. The 1.6 mm guide wire was placed identically “high and inside,” and the manufacturer's cannulated drills (3.2/5.0 mm) and taps (4.5/6.5 mm) were used in a similar technique. In the HCS group, the compression sleeve was used to countersink the screw head.

The small matched pair specimens (6/10) that received a 4.5 mm JFXS on one side received a 4.5 mm HCS screw on the other side accordingly. Vice versa we proceeded with larger specimens (4/10) and implanted 6.0 mm JFXS or 6.5 mm HCS screws.

### Biomechanical Test Setup

**Figure 2:**
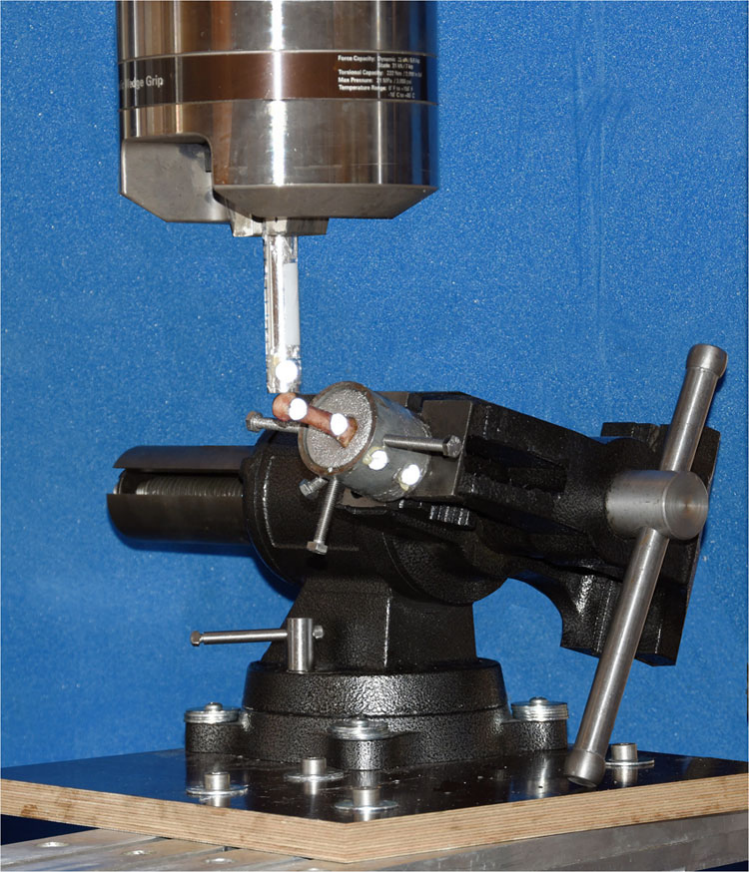
Biomechanical test setup. The potted fifth metatarsal specimen was fixed into a machine vice on an adjustable platform. A metal rod attached to the loading frame was used for force transmission in a plantar to dorsal direction. Light‐reflecting hemispherical markers were glued onto the distal part of the Jones fracture specimen and onto the pot and rod for kinematic tracking. [Color figure can be viewed at wileyonlinelibrary.com]

An experimental setup was designed to simulate post‐operative in vivo load conditions after surgical Jones fracture treatment. The fifth metatarsal specimens were potted with their proximal aspects in Wood's metal in 40 mm diameter custom built steel cups. The Jones fracture site was kept outside of the molding material. Wood's metal is a moldable bonding material for biomechanical testing and it was proven to be superior compared with polymethylmethacrylate (PMMA),^30^ which is commonly used in similar biomechanical setups. The exposed screw head was covered with modeling clay to isolate it from the embedding material. Care was taken to cover just the proximal part of the bone and not the fracture site with the molding material. The steel cups were mounted in a machine vice that is fixed to an adjustable platform. The fifth metatarsal was aligned such that the plantar surface was directed upwards with a slight angulation of 7–10° plantarflexion, mimicking the fifth metatarsal stacking angle.^21^ A fixed self‐leveling horizontal laser beam (PLL 360; Robert Bosch GmbH, Leinfelden‐Echterdingen, Germany) and a goniometer were used for alignment verification during mounting. The biomechanical testing for this study was performed with an 858 Mini Bionix® (MTS® Systems Corporation, Eden Prairie, MN). The 858 Mini Bionix® is a servo‐hydraulic test frame, consisting of a loading frame (MTS® 858; Eden Prairie) with a stroke main actuator driven by a hydraulic pump unit (MTS® 505.11 silent flow; Eden Prairie). A metal rod was used for force transmission onto the metatarsal (Fig. 2). The force measurement transducer was integrated into the 858 Mini Bionix® testing system. The uncertainty in measurement for force of the system is 1%. The load was recorded during testing continuously at a sampling frequency of 60 Hz. An opto‐electronic motion capture system (Smart‐E; BTS Bioengineering, Milan, Italy) with four cameras was used during the loading process at a sampling rate of 120 Hz for kinematic measurements. Two 5 mm hemispherical markers were attached to the proximal aspect of the fractured metatarsal (steel cup), two markers were placed at the distal metatarsal and one marker was fixed to the rod applying the load on the metatarsal head. Epoxy glue was used for marker fixation. In addition, biomechanical testing was video recorded (D7200; Nikon, Tokyo, Japan).

### Cyclic Partial Weight‐Bearing Model

The load was applied at the plantar aspect of the fifth metatarsal head. The specimens were loaded in compression from plantar to dorsal in a force‐controlled manner at 3 N/s up to 12 N. On the supposition that early post‐operative mobilization would involve a partial weight‐bearing regimen, 12 N has been chosen as the peak load. This load represents one‐half of the load to which the head of the fifth metatarsal is subjected during the normal push‐off phase of gait.^26^, ^33^ This position was held for 5 s. The machine displacement and angulation of the distal bone fragment was measured and unloaded at the same rate. The specimen was loaded with a cyclic load mean of 12 N at 0.5 Hz for 1000 cycles. The number of load cycles was chosen based on the loading rate for a physiologically normal lower limb, which is approximately 5000 cycles per day. One thousand cycles per day was assumed to realistically simulate post‐operative loading in an active patient.^26^, ^34^, ^35^ The load–displacement curves for each construct were filtered using the digital Savitzky–Golay filter before analysis.^36^ The ascending linear region of the load–displacement curve was used to measure the stiffness (slope), which was expressed as N/mm. Data were recorded at following loading cycles: 1, 10, 100, 200, 300, 400, 500, 600, 700, 800, 900, and 1000.

### Ultimate Load Model and Modes of Failure

After completion of cycle 1000, an ultimate load test was performed with the same force rate as described above. The failure of the construct was defined by exceeding 10° of interfragmentary angulation or until gross construct failure occurred (i.e., screw failure or bone fracture). In constructs that failed before loading cycle 1000, the load applied at the time of failure was defined as the ultimate load.

Modes of failure were classified after completion of the load to failure testing. Each specimen was liberated from Wood's metal fixation and inspected by two orthopedic surgeons (M.W. and R.S.). The mode of failure was determined upon agreement of both investigators after visual inspection of the specimens and video recordings. Standardized photographs were taken of each construct failure (D7200; Nikon).

### Statistical Analysis

Statistical analyses were performed using SPSS 20.0 for Windows (SPSS Inc, Chicago, IL), and the level of significance was defined as α < 0.05. Descriptive data were reported as means with standard deviation. All data showed a normal distribution in Kolmogorov–Smirnov test.

Paired samples *t* test was used to determine the significance of difference in stiffness (slope), displacement, angulation, and ultimate load between the experimental groups at each loading cycle.

Intergroup differences between screw diameters were analyzed using independent *t* test.

Pearson product‐moment correlation coefficient was calculated in order to investigate the relationship between BMD and angulation, stiffness, and ultimate load.

## RESULTS

### Stiffness (Slope of the Load–Displacement Curve)

There was no significant difference in stiffness and machine displacement between fifth metatarsals fixed with the solid Jones Fracture‐specific screw (JFXS group) or the cannulated headless compression screw (HCS group) at any point during the cyclic loading (stiffness:0.324 ≤ *p* ≤ 0.986; displacement: 0.131 ≤ *p* ≤ 0.635). The mean stiffness in the JFXS group and in the HCS group reached 40.6 ± 7.5 and 40.3 ± 13.5 N/mm at load cycle 1000, respectively (*p* = 0.928) (Table 1).

**Table 1:** Stiffness in Solid Fracture‐Specific Screws (JFXS) and Cannulated Headless Compression Screws (HCS) During Cyclic Loading

		JFXS		HCS		
Cycle Number	Count (Pairs)	Mean (N/mm)	SD	Mean (N/mm)	SD	*p* Value
1	10	22.9	23.5	16.1	16.9	0.324
10	10	39.4	16.2	37.2	18.8	0.744
100	9	39.8	8.8	37.5	11.3	0.474
200	9	36.0	15.0	38.5	12.2	0.678
300	9	40.5	7.7	39.2	12.9	0.749
400	9	40.0	7.6	39.4	12.0	0.903
500	9	40.3	7.6	39.1	12.0	0.712
600	9	40.1	8.0	39.7	12.6	0.926
700	9	40.4	7.7	40.0	13.2	0.895
800	9	40.9	7.5	40.3	13.4	0.855
900	9	40.6	7.3	40.6	13.2	0.986
1,000	9	40.6	7.5	40.3	13.5	0.928

Means with standard deviations of stiffness (slope) of Jones Fracture constructs during cyclic loading.

### Angulation

**Figure 3:**
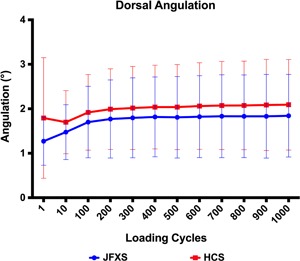
Interfragmentary angulation (°) throughout cyclic loading for solid Jones fracture‐specific screws (JFXS—blue) and cannulated headless compression screws (HCS—red). The groups did not differ in statistical significance (0.238 ≤ *p* ≤ 0.600). [Color figure can be viewed at wileyonlinelibrary.com]

All constructs in the JFXS group survived 1000 loading cycles without exceeding 10° of dorsal angulation. One construct in the HCS group failed between the 1st and the 10th cycle with an interfragmentary angulation of 21°. This specimen had the lowest BMD value among all tested feet (0.169 g/cm^2^) and it sustained a plantar metatarsal shaft fracture. According to preliminary failure criteria, we found a construct survival of 100% in the JFXS group and 90% in the HCS group. Matched pair analysis showed no statistically significant difference between the screw groups (0.238 ≤ *p* ≤ 0.600) (Table 2 and Fig. 3).

**Table 2:** Interfragmentary Angulation

Group	Angulation 1st Load Cycle	Angulation 1000th Load Cycle	Angulation Increase From 1st to 1000th Load Cycles
JFXS	1.3° (0.5)	1.8° (0.9)	38.4%
HCS	1.8° (1.4)	2.1° (1.0)	16.7%
Mean difference % (*p* value)	38.4% (*p* = 0.238)	16.7% (*p* = 0.311)	

Means with standard deviations in parenthesis of interfragmentary angulation between the proximal and distal aspect of Jones fracture specimens during cyclic loading. Differences in angulation increase and intergroup differences are given in percentages with corresponding *p* values.

HCS, headless compression screws; JFXS, Jones fracture‐specific screw.

### Ultimate Load

**Figure 4:**
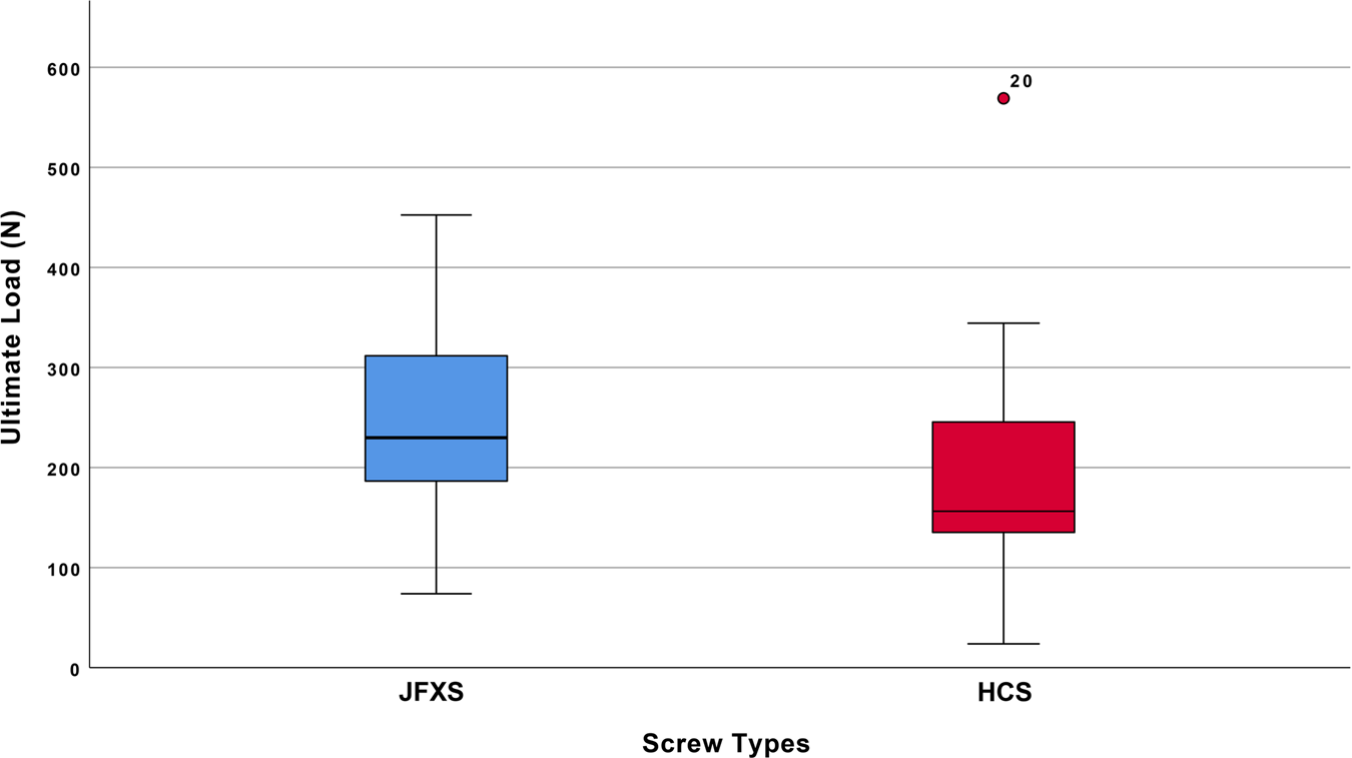
Box plots representing the ultimate load to failure, in Newton, per screw. The horizontal line indicates the median, the box extends from the 25th to the 75th percentile, and the bars indicate the largest and smallest observed value. [Color figure can be viewed at wileyonlinelibrary.com]

The ultimate load until an occurrence of failure reached 236.9 ± 107.8 N in the JFXS group compared with 210.8 ± 150.7 N in the HCS group. Intergroup difference was not statistically significant (*p* = 0.429) (Fig. 4).

When comparing screw diameters we found 202.1 ± 91.5 N load until failure in 4.5 mm and 289.3 ± 121.8 N in 6.0 mm solid JFXS constructs (*p* = 0.230). The HCS diameters differed statistically significantly with a mean of 133.5 ± 59.1 N in 4.5 mm screws and 326.9 ± 180.0 N in 6.5 mm screws concerning the ultimate load (*p* = 0.037).

### Mode of Failure

The most common mode of failure in HCS constructs was proximal screw head cut out (*n* = 6, 60%), followed by loosening of the screw head (*n* = 3, 30%). The screw head cut out was defined as a sharp cut through the screw head in the proximal aspect of the fifth metatarsal. The screw was still rigidly embedded in the bone, but not at the original place of insertion. In contrast to a cut out of the screw head, we also found loosening of the screw head in some specimens. The screw head was loose in the proximal bone with rather a bony defect around the head.

**Figure 5:**
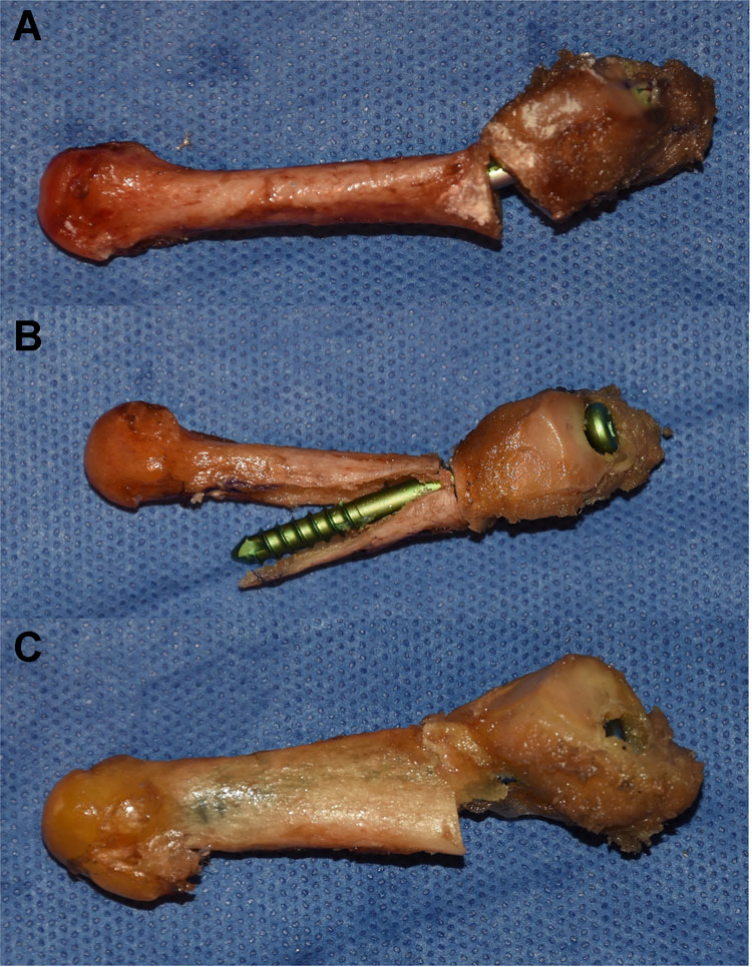
Exemplary modes of failure during load to failure testing. (A) Screw head loosening and consecutive bending of the screw. (B) Diaphyseal shaft fracture with plantar dislocation of the screw. (C) Screw head cut out. [Color figure can be viewed at wileyonlinelibrary.com]

In JFXS constructs metatarsal shaft fracture was the most observed mode of failure (*n* = 4, 40%), followed by screw head cut out (*n* = 3, 30%). One JFXS construct showed a rotational instability at the proximal aspect of the fifth metatarsal. In this construct, a 4.5 mm screw was used and the specimen had a low BMD of 0.208 g/cm^2^ (Table 3 and Fig. 5). Screw failure in terms of a bending of the screw occurred in five JFXS constructs and in five HCS constructs (50% JFXS vs. 50% HCS). The screw bending was seen more often in small diameter screws. No screw breakage was observed in the tested constructs (Table 3).

**Table 3:** Mode of Failure and Screw Bending

Screw Type, Screw Diameter, (Count)		Screw Head Cut Out	Shaft Fracture	Screw Head Loosening	Rotational Instability	Screw Bending (%)
JFXS	4.5 mm (6)	1	2^a^	2	1	2 (33)
	6.0 mm (4)	2	2	0	0	3 (75)
HCS	4.5 mm (6)	3	1	2	0	5 (83)
	6.5 mm (4)	3	0	1	0	0 (0)

The mode of failure during the ultimate load to failure testing listed by screw type and diameter is outlined. Additional screw failure due to bending of the screw is indicated.

HCS, headless compression screws; JFXS, Jones fracture‐specific screw.

^a^ One specimen failed preliminarily due to metatarsal shaft fracture, which occurred during the 1st and 10th loading cycle.

### Bone Mineral Density

The mean BMD in the JFXS group was 0.389 g/cm^2^ (±0.13, range 0.208–0.633) and 0.399 g/cm^2^ (±0.15, range 0.169–0.649) in the HCS group. There was no statistically significant difference between the groups (*p* = 0.839) and BMD did not correlate with age (*R* = −0.226, *p* = 0.338). There was a statistically significant difference in BMD between male and female specimens. The mean BMD in male specimens reached 0.446 g/cm^2^ compared with 0.315 g/cm^2^ in female specimens (*p *= 0.032). BMD showed a positive correlation with the pooled ultimate load (*R* = 0.580, *p* = 0.007) for all constructs. It correlated negatively with angulation (angulation at first loading cycle: *R* = −0.676, *p* = 0.003), which was significant for every load cycle among all tested constructs (angulation at cycle 10: *R* = −0.552, *p* = 0.002; cycle 100–1,000: *p* ≤ 0.001). We also found a positive correlation of BMD with the stiffness during the initial phase of cyclic loading (cycle 1: *R *= 0.549, *p* = 0.012; cycle 10: *R* = 0.527, *p* = 0.017). The later loading cycles showed no significant correlation with the stiffness groups (0.121 ≤ *p* ≤ 0.287).

## DISCUSSION

The Jones fracture is a common sports injury, especially in professional athletes, and fast return to sports and competition is the primary aim of surgery.^4^, ^5^, ^8^, ^10^ Clinical studies already proved the safety and efficacy of intramedullary screw fixation in competitive athletes who sustained proximal fifth metatarsal fractures (Zones II and III).^4^, ^5^, ^8^, ^10^, ^12^, ^15^, ^17^, ^37^ Nevertheless, refracture, screw failure, non‐union, or screw head discomfort are frequent problems in this group of high‐demand patients.^2^, ^3^, ^4^, ^5^, ^6^, ^8^, ^10^, ^16^, ^23^, ^24^ Therefore, implant choice is still a matter of concern. In the present study, a biomechanical comparison of a Jones fracture‐specific solid partially threaded titanium screw with a small low‐profile head and a conventional countersinkable cannulated partially threaded titanium screw has been performed. The Jones fracture constructs were loaded for 1000 cycles with a plantar to dorsal‐oriented load force followed by ultimate load to failure testing. Angulation, stiffness, ultimate load, and modes of failure were recorded and analyzed.

We found higher ultimate loads during load to failure testing for the Jones fracture‐specific screw (236.9 ± 107.8) compared with the headless compression screw (210.8 ± 150.7 N) (*p* = 0.429) though results were not statistically significant. In terms of stiffness (slope of the load–displacement curve) and angulation, we observed no difference in paired analysis between the JFXS and the HCS. Screw head cut out and screw head loosening comprised the most common modes of failure. This mode of failure was observed more frequently in HCS than in JFXS. We also observed higher initial angulation during cyclic loading in the HCS group in comparison with the JFXS group (mean difference of 38.4%; *p* = 0.238). One possible explanation for these observations could be that the engaging threads of the HCS head cut through the cancellous bone in the metaphysis of the fifth metatarsal and therefore the screws loosen easier during loading. Orr et al.^26^ compared fully threaded tapered variable pitch screws (Acutrak, Acumed, Hillsboro, OR) to conventional partially threaded solid screws (Synthes, Monument, CO) in a cyclic loading Jones fracture fixation model. They also observed higher angulation during cyclic loading in the tapered variable pitch screw group, which could be due to a similar loosening mechanism. Regarding BMD we found a positive correlation with ultimate load and a negative correlation with angulation throughout cyclic loading. Initial stability (cycles 1–10) in terms of stiffness was also positively correlated with BMD. These results implicate that the stability of intramedullary screw fixation is diminished in individuals with low BMD. In terms of screw diameter, our results are in concordance with the available biomechanical literature that showed advantageous biomechanical properties in larger diameter screws due to increased screw pullout strength.^12^, ^27^, ^38^ However, these potential biomechanical advantages have to be proven in clinical studies.

Several studies evaluated different fixation methods for surgical Jones fracture treatment in various biomechanical test setups.^2^, ^5^, ^8^, ^12^, ^14^, ^15^, ^16^, ^17^, ^25^, ^26^, ^27^, ^31^, ^38^, ^39^, ^40^, ^41^ Recent attention has been focused on Jones fracture‐specific implants such as specifically designed screws and plates.^2^, ^4^, ^16^, ^20^, ^31^, ^32^, ^40^, ^41^ Latest biomechanical studies revealed contradictory results comparing Jones fracture‐specific screw to plate fixation.^8^, ^10^, ^14^, ^16^, ^17^, ^23^, ^24^, ^25^, ^26^, ^27^, ^28^, ^40^, ^41^ The comparison of two intramedullary screw constructs was technically more appealing to us because the clinical use of percutaneous screw placement is generally accepted as gold standard treatment. With our biomechanical test setup we tried to simulate an early post‐operative partial weight‐bearing regimen by cyclic plantar to dorsal loading. The load to failure test aims to simulate maximum applied forces at the time of full return to competitive sports. Similar test setups to approximate weight‐bearing have been carried out in hallux surgery and Jones fracture studies.^25^, ^26^, ^28^, ^31^, ^32^, ^34^, ^35^

Practically, every biomechanical study has to face the same inherent limitations such as a limited specimen number, risking a type II error, and a simplified simulation of in vivo biomechanics. In order to account for the potential drawback of high donor age, which does not represent the typical athletic population, we used a matched pair study design and performed BMD evaluation prior to biomechanical testing, showing no difference between the specimen groups. Another concern is the diameter of the screws, which were used in this study. Among available screws a substantial discontinuity exists in their dimensional range. The solid Jones Fracture Screw (Arthrex Inc., Naples, FL) is available in the diameters 4.5, 5.5, and 6.0 mm. The commonly obtainable and for multiple fracture fixations applicable cannulated Headless Compression Screw (HCS) is produced in diameters 4.5 and 6.5 mm. In order to compare equal screw sizes in this matched pair study, we used 4.5 mm JFXS or HCS in smaller specimens and compared a 6.0 mm JFXS to a 6.5 mm HCS fixation in larger specimens. We are aware of this mismatch. Nevertheless, this conflict reflects the surgeon's dilemma in the clinical routine, which is also limited by available implants and diameters.

## CONCLUSION

Data of the present Jones fracture fixation model shows that both screw constructs (solid fracture‐specific screws versus conventional cannulated headless compression screws) provide equal ultimate loads and stiffness. In addition, low BMD seems to be related to a debilitated primary stability and diminished ultimate load in intramedullary Jones fracture fixation.

## AUTHORS’ CONTRIBUTION

M.W.: Designing the study, preparing the specimens, performing the experiments, analyzing the data, statistical analysis, figure design, drafting, revising, and approving the manuscript. E.B.: Performing the experiments, drafting, revising, and approving the manuscript. L.H.: Preparing the specimens, revising and approving the manuscript. M.F.K.: Statistical analysis, figure design, revising and approving the manuscript. G.B.: Preparing the specimens, performing the experiments, drafting and approving the manuscript. S.Z.: Radiological investigations, approving the manuscript. R.W.: Revising and approving the manuscript. R.S.: Designing the study, surgical procedure, revising and approving the manuscript. Each author represents that he has read and approved the final manuscript.
